# Carbapenemase-producing Enterobacterales isolated from hospital sinks: molecular relationships with isolates from patients and the change in contamination status after daily disinfection with sodium hypochlorite

**DOI:** 10.1017/ash.2024.94

**Published:** 2024-06-04

**Authors:** Yoshiaki Shikama, Chiemi Yokoya, Akira Ohara, Megumi Yamashita, Yuichi Shimizu, Tomoyuki Imagawa

**Affiliations:** 1 Infection Control and Prevention Service, Kanagawa Children’s Medical Center, Yokohama, Japan; 2 Department of Infectious Disease and Immunology, Kanagawa Children’s Medical Center, Yokohama, Japan; 3 Department of Clinical Laboratory, Kanagawa Children’s Medical Center, Yokohama, Japan; 4 Department of Pharmacy, Kanagawa Children’s Medical Center, Yokohama, Japan

## Abstract

**Objective:**

This study aimed to investigate the contamination status of hospital sinks with carbapenemase-producing Enterobacterales (CPE), the efficacy of daily cleaning with sodium hypochlorite, and the relationships between CPEs isolated from contaminated sinks and patients.

**Design:**

Pre/postintervention surveys of the CPE-contaminated sinks.

**Setting:**

Hospital wards including pediatric intensive care unit in a children’s hospital.

**Participants:**

Consenting CPE-colonized patients admitted between November 2018 and June 2021 in our hospital.

**Methods:**

Environmental culture of 180 sinks from nine wards in our hospital was performed three times with an interval of 2 years (2019, 2021, 2023). Molecular typing of the isolated strains from the sinks and patients was performed. After the first surveillance culture, we initiated daily disinfection of the sinks using sodium hypochlorite.

**Results:**

Before the intervention, we detected 30 CPE-positive sinks in 2019. After the intervention with sodium hypochlorite, we observed a substantial decline in the number of sinks contaminated with CPE; 13 in 2021 and 6 in 2023. However, the intervention did not significantly reduce the number of CPE-contaminated sinks used for the disposal of nutrition-rich substances. The CPE isolates from the patients and those from the sinks of the wards or floors where they were admitted tended to have similar pulse-field gel electrophoresis patterns.

**Conclusion:**

Contaminated sinks could be reservoirs of disseminating CPE to the patients. Daily disinfection of sinks with sodium hypochlorite may be effective in eliminating CPE, although the effect could be weaker in sinks with a greater risk of contact with nutrition-rich substances.

## Introduction

Carbapenem antimicrobials have a broad spectrum of antimicrobial activity which exceeds that of most other antimicrobial classes. They are used for the treatment of severe infections caused by Gram-negative rods that do not respond to other antimicrobials.^1^ Enterobacterales that are resistant to at least one of the carbapenem antibiotics are called carbapenem-resistant Enterobacterales (CRE). A subset of CRE can produce carbapenem-hydrolyzing enzymes, called carbapenemase-producing Enterobacterales (CPE). Because of the limitations of existing therapeutic options, CPE infections are associated with high morbidity and mortality rates. Moreover, they can spread their resistance-associated genetic elements easily and are involved in many healthcare-associated outbreaks.^2^ Therefore, CPE infections are a major threat to public health worldwide and are considered “priority pathogens” by the World Health Organization for which new antibiotics are urgently needed.^3^ CPE outbreaks have been reported from most parts of the world, and hospital water environments have become a common reservoir of CPE in recent years.^4,5^ CPE forms biofilm on the surface of sink drain tubes and cannot be readily eliminated even after cleaning and disinfection.^6^

Herein we report the CPE contamination status of sinks in the hospital wards, relationships between CPE strains isolated from patients and sinks, and the effects of daily cleaning and disinfection of sinks using sodium hypochlorite.

## Materials and methods

### Setting and surveillance culture

Kanagawa Children’s Medical Center is a 430-bed tertiary-care hospital for children located in Yokohama, Japan. Its main tower comprises outpatient clinics and nine inpatient wards, including a 10-bed pediatric intensive care unit (PICU), 14-bed high-care unit, and 30-bed cardiology ward. The PICU consists of 10 beds for medical and surgical patients, with approximately 80% of annual admissions for perioperative management of cardiovascular surgery. The PICU, high-care unit, and cardiology ward are located on the third floor of the main tower. The fourth floor consists of three surgical wards (4W, 4E, and 4S), and the fifth floor has three medical wards (5W, 5E, and 5S).

Environmental surveillance cultures of the sinks were implemented in February 2019, 2021, and 2023. A sample from one sink was obtained using one sterile cotton swab, wiping the surface of the sink bowl and drain outlet. Deeper zones of sink drains were not wiped. In addition to sinks used for handwashing, those used for other purposes (e.g., milk preparation and sanitary waste treatment) were included in the surveillance. Between April 2020 and June 2021, 19 patients from the PICU and cardiology ward were newly recognized as CPE-positive. In addition to the surveillance culture mentioned above, environmental sampling was repeatedly conducted during the outbreak period. We collected swab samples from sinks, faucets, and high-touch surfaces, including beds, monitors, pumps, and computer keyboards located around CPE-positive patients.

### Microbiological methods

Surveillance rectal and environmental samples were obtained using sterile cotton swabs. The swab samples were streaked onto Chromagar mSuperCARBA plates (Kanto Chemical Co., Inc., Tokyo, Japan) and incubated overnight at 35°C in ambient air. Broth enrichment was not performed. Suspicious colonies were identified using matrix-assisted laser desorption/ionization time-of-flight mass spectroscopy (VITEK MS; bioMérieux, Lyon, France). To detect carbapenemase, we used Cica-βtest MBL and MASTDISCS combi Carba plus disc system (MD).^7^ Carbapenemase genes were identified using multiplex polymerase chain reaction, according to the methods described by Watahiki et al.^8^ Molecular characterization of the outbreak and environmental isolates was performed using pulse-field gel electrophoresis (PFGE). Genomic DNA of *Pantoea* sp., *Enterobacter cloacae* complex, and *Klebsiella oxytoca* were processed using *Xba* I, *Spe* I, and *Xba* I, respectively. The strains were evaluated using the Bionumerics software system (version 7.6.3; Applied Maths, Sint-Martens-Latem, Belgium).

### Cleaning and disinfecting sinks

Before the first environmental cultures were obtained in 2019, we only washed the sinks in hospital wards with neutral detergent. After contamination of several sinks in the wards with CPE, we started to clean all the sinks with foam-type 0.1% sodium hypochlorite every 1–2 days. To clean the sinks, appropriate personal protective equipment, such as eye guards, gloves, and disposable aprons, were worn. Then, foam-type sodium hypochlorite was sprayed directly into sink bowls, drain holes, and faucets. Next, disposable cotton gauze was used to wipe foam from outside to inside, followed by drain holes. Finally, the sinks were rinsed with water.

### Statistical analysis

All statistical analyses were performed using EZR (Saitama Medical Center, Jichi Medical University, Saitama, Japan), a graphic user interface for R (The R Foundation for Statistical Computing, Vienna, Austria).^9^

We performed McNemar’s chi-square test to evaluate the effect of daily cleaning with bleach on the number of CPE-positive sinks.

## Results

### Detection of new CPE carriers and first surveillance in 2019

**Figure 1.**
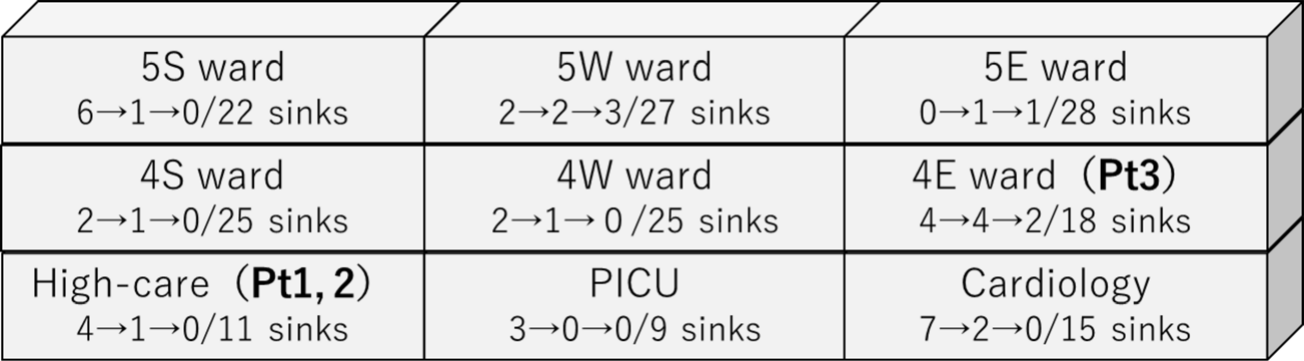
Numbers of Carbapenemase-producing Enterobacterales (CPE)-positive sinks in each ward (years 2019→2021→2023). The isolated CPE strains in each year are listed in Supplementary Table S1.

Between November 2018 and January 2019, CPE infections were detected in three hospitalized patients (Table 1, Patients 1–3). Patients 1 and 2 were admitted to the high-care unit, whereas patient 3 was admitted in the 4E ward (Figure 1). They had all been hospitalized for longer than 1 month. The CPE species detected in patient 1 was *Pantoea* sp., patient 2 was *K. oxytoca* and *Citrobacter freundii* complex, and patient 3 had *Klebsiella pneumoniae*. All four CPE isolates produced IMP-11. CPE was not detected from the other patients or the environment of CPE-positive patients. Notably, some sinks near patients 1 and 2 were positive for CPE, but the isolated species were not always the same as those detected from the patients.

**Table 1.** List of patients carrying Carbapenemase-producing Enterobacterales (CPE). Patients marked with asterisk had never been admitted to the cardiology ward before they were found to carry CPE

Patient No.	Gender	Age	Ward	Organism	Carbapenemase	Date detected (Month-Year)
1	M	2 y 5 m	High care	*Pantoea sp.*	IMP-11	Nov-18
2	F	0 y 6 m	High care	*K. oxytoca* *C. freundii complex*	IMP-11	Nov-18
3	F	9 y 5 m	4E	*E. cloacae* complex	IMP-11	Jan-19
4*	F	0 y 0 m	PICU	*E. cloacae* complex	IMP-1	Apr-20
5*	F	0 y 0 m	PICU	*E. cloacae* complex	IMP-1	Apr-20
6*	M	0 y 1 m	PICU	*E. cloacae* complex	IMP-1	Apr-20
7*	F	0 y 1 m	PICU	*E. cloacae* complex	IMP-1	Apr-20
8	M	0 y 0 m	Cardiology	*E. cloacae* complex	IMP-1	Jul-20
9	M	0 y 5 m	Cardiology	*E. cloacae* complex	IMP-1	Aug-20
10	M	0 y 3 m	Cardiology	*E. cloacae* complex	IMP-1	Aug-20
11	F	0 y 1 m	PICU	*E. cloacae* complex	IMP-1	Aug-20
12	M	0 y 3 m	Cardiology	*E. cloacae* complex	IMP-1	Aug-20
13	F	0 y 1 m	Cardiology	*E. cloacae* complex	IMP-1	Aug-20
14*	F	0 y 1 m	PICU	*E. cloacae* complex	IMP-1	Aug-20
15	M	0 y 2 m	Cardiology	*E. cloacae* complex	IMP-1	Sep-20
16*	M	0 y 2 m	PICU	*E. cloacae* complex	IMP-1	Nov-20
17*	F	0 y 0 m	PICU	*E. cloacae* complex	IMP-1	Mar-21
18*	M	0 y 0 m	PICU	*E. cloacae* complex	IMP-1	Apr-21
19	M	0 y 11 m	Cardiology	*E. cloacae* complex	IMP-1	May-21
20	F	0 y 6 m	Cardiology	*E. cloacae* complex	IMP-1	Jun-21
21	M	0 y 9 m	Cardiology	*E. cloacae* complex	IMP-1	Jun-21
22	F	0 y 9 m	PICU	*E. cloacae* complex	IMP-1	Jun-21

This suggested that additional sinks might be contaminated with CPEs. We conducted environmental surveillance cultures of the sinks of nine wards in the main tower. Environmental samples from the sinks were collected, seeded onto CPE selection agar plates, and subjected to identification and susceptibility testing. The results are shown in Figure 1. Of the 180 sinks from nine wards screened, 30 were contaminated with CPE. The isolated strains were *Pantoea* sp. (*n* = 20), *E. cloacae* complex (*n* = 5), *K. oxytoca* (*n* = 2), *Pseudescherichia vulneris* (*n* = 2), and *C. freundii* complex (*n* = 1). Three isolates had carbapenemase gene *bla*_IMP-1_ (one *E. cloacae* complex and two *P. vulneris*), whereas the others had *bla*_IMP-11_ (Supplementary Table S1).

**Figure 2.**
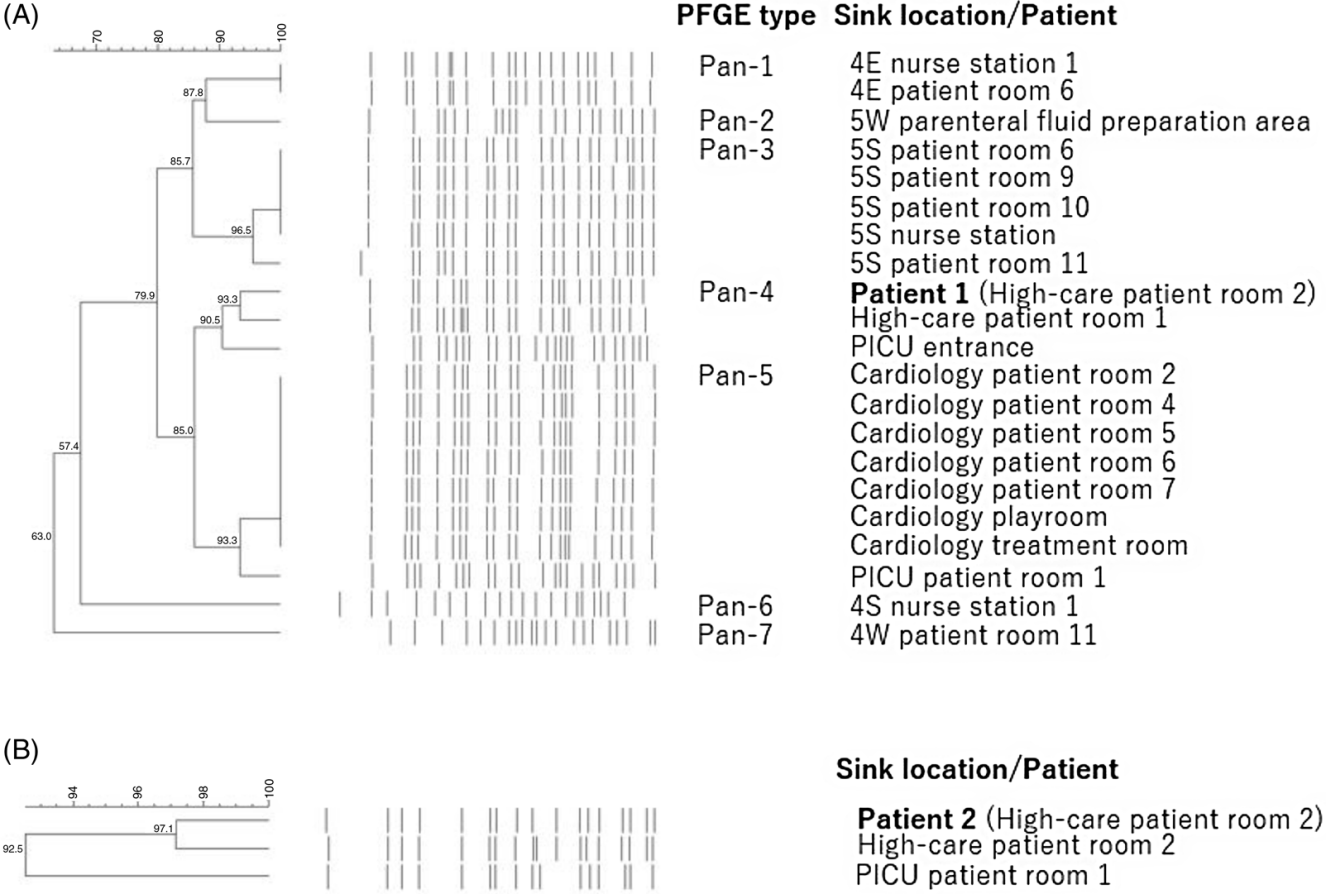
Pulse-field gel electrophoresis patterns and phylogenic trees of Carbapenemase-producing Enterobacterales isolated from sinks and patients in 2018–2019. A: *Pantoea* sp. B: *Klebsiella oxytoca*.

As shown in Figure 2, the isolated *Pantoea* spp. belonged to seven PFGE groups, and the isolates from the same wards or floors tended to have similar PFGE patterns. *Pantoea* spp. strains isolated from patient 1 and a sink located in a different room of the ward where he was admitted had similar PFGE patterns (>90% similarity). Similarly, the *K. oxytoca* strain isolated from patient 2 had similar PFGE patterns as those from a sink of the room where she was admitted, suggesting horizontal transmission.

The surveillance culture results urged us to take actions to prevent the CPE spread from contaminated sinks. We started to disinfect the sinks with foam-type 0.1% sodium hypochlorite, instead of neutral detergent, once every 1–2 days.

### Carbapenemase-producing E. cloacae complex outbreak and second surveillance in 2021

**Figure 3.**
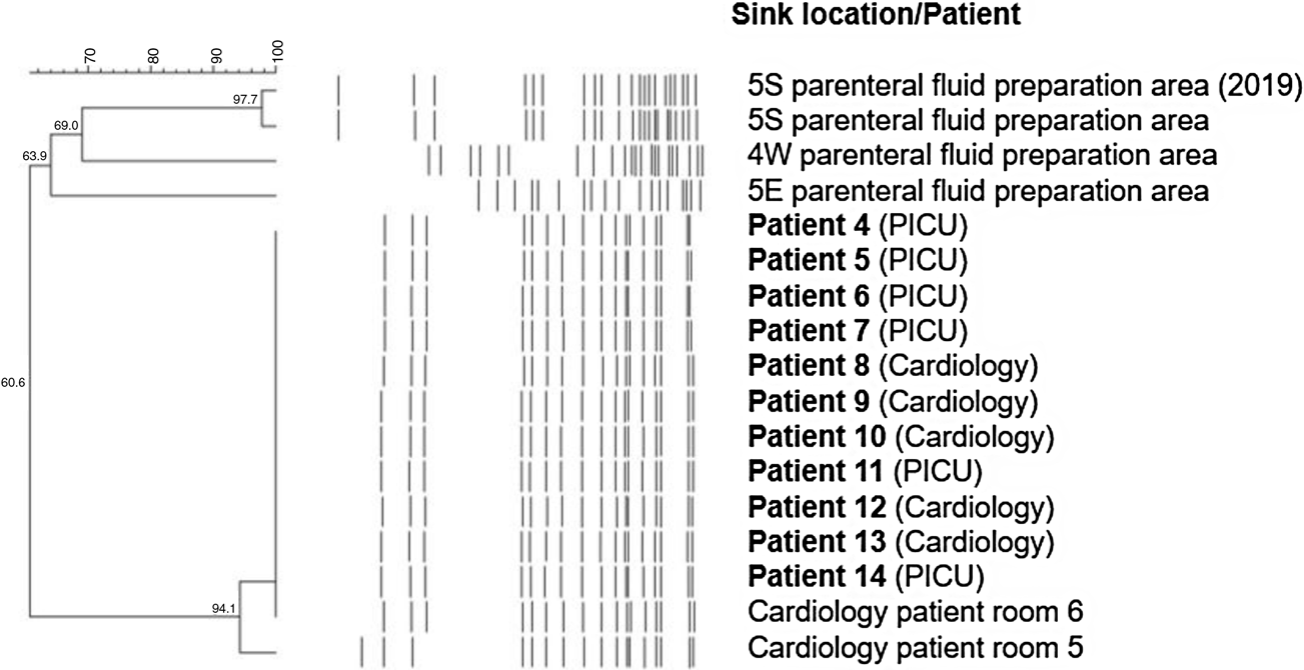
Pulse-field gel electrophoresis patterns and phylogenic trees of IMP-1-producing *Enterobacter cloacae* complex isolated from sinks and patients in 2021.

Between April 2020 and June 2021, 19 patients were newly found to be CPE carriers (Table 1, Patients 4–22). IMP-1-producing *E. cloacae* complex was isolated from all patients. These patients were infants with congenital heart diseases and had been hospitalized for long periods of time, transferring between the cardiology ward and PICU. Although swabs from dry surfaces in the PICU and cardiology ward did not yield any *E. cloacae* complex isolates, swabs from the sinks of two patient rooms in the cardiology ward yielded *E. cloacae* complex that produced IMP-1. These sinks (rooms 5 and 6; Supplementary Table S1) were not contaminated with CPE-producing *E. cloacae* complex in 2019. The PFGE patterns of the isolates from the patients showed similar patterns to the sinks, suggesting a relationship among them (Figure 3; isolates from 11 of 19 patients are shown: Patients 4–14). However, eight of 19 patients were found to be CPE-positive without being admitted to the cardiology ward (Table 1, marked with asterisk), and the two rooms had been used for cohorting of CPE-positive patients since June 2020. Therefore, the sinks might be contaminated after some CPE-positive patients were admitted.

Along with the cardiology ward and PICU, the sinks from the other seven wards were re-inspected for CPE in February 2021 using similar methods as those used in 2019. Of the 180 sinks, 13 were contaminated with CPEs, including the two aforementioned sinks located in the cardiology ward (Figure 1, Supplementary Table S1). The isolated strains were *E. cloacae* complex (*n* = 6), *K. oxytoca* (*n* = 3), *C. freundii* complex (*n* = 3), and *Pantoea* sp. (*n* = 1). Four *E. cloacae* complex isolates produced IMP-1, whereas the other isolates produced IMP-11.

Considering the environmental culture results, horizontal transmission due to insufficient hand hygiene was the main cause of the IMP-1-producing *E. cloacae* complex outbreak. To perform appropriate hand hygiene, handwashing sinks in the PICU, high-care unit, and cardiology ward were replaced with those with deeper bowls. In addition, to prevent bacterial feeding in the drain, discarding excess milk and hyperalimentary fluid down the sinks was prohibited.

### Third surveillance in 2023 and comparison with previous data

After the outbreak in 2020–2021 ceased, no patients were newly proven to be a CPE carrier. To determine whether our strategy had further decreased the number of CPE-contaminated sinks, we implemented a third round of CPE surveillance in February 2023. Six sinks were found to be CPE-positive (Figure 1, Supplementary Table S1). *E. cloacae* complex was detected from five sinks, and *K. oxytoca* from one sink. Four isolates of *E. cloacae* complex produced IMP-1, whereas one isolate of *E. cloacae* complex and the isolate of *K. oxytoca* produced IMP-11.

**Figure 4.**
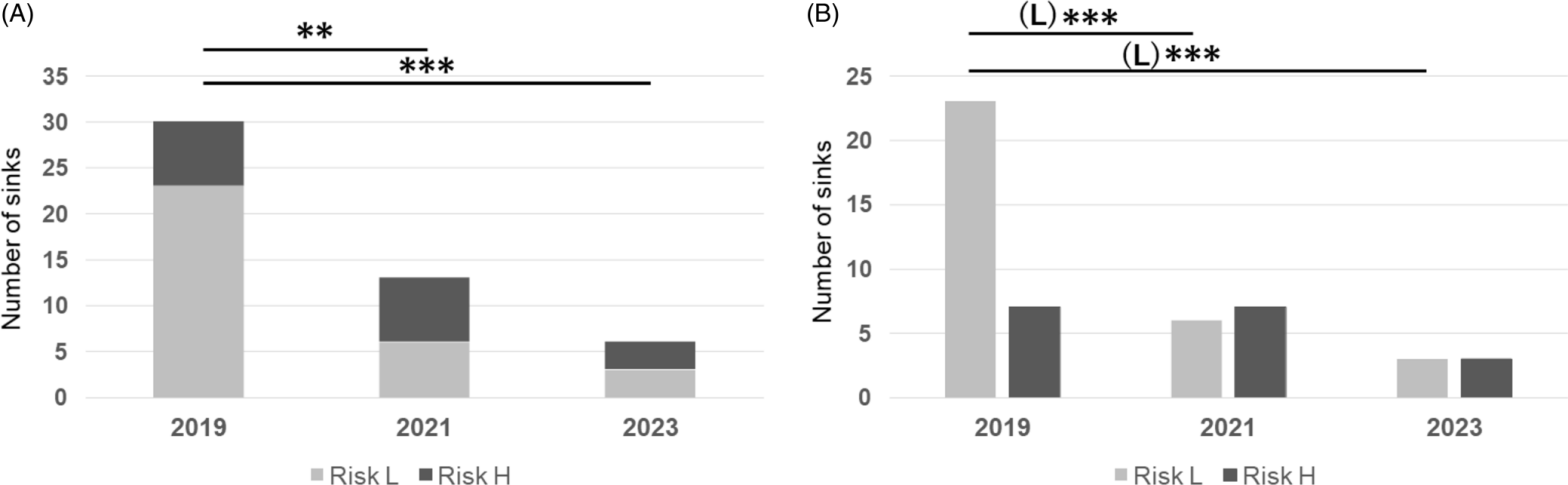
Number of sinks contaminated with Carbapenemase-producing Enterobacterales examined in 2019, 2021, and 2023. A: total numbers, B: numbers of sinks classified by the risk of contact with nutrition-rich substances; Risk L (low), Risk H (high). **: *p* < .01, ***: *p* < .001.

Some sinks had been used not only for handwashing, but also for the disposal of excess milk and hyperalimentary fluid. Because increased nutrient availability can support biofilm growth, we compared the CPE elimination rate between the sinks used for handwashing only (Risk L) and those used for discarding nutrition-rich substances (Risk H). Sinks located in nurse stations, treatment rooms, patient rooms, and restrooms were assigned Risk L, whereas those used for milk preparation and dish washing, located in the parenteral fluid preparation area and dirty utility rooms, were assigned Risk H. A comparison of the environmental culture results from 2019, 2021, and 2023 revealed that the number of CPE-positive Risk L sinks was significantly decreased. However, no significant decrease was observed in the number of Risk H sinks (Figure 4).

## Discussion

Given the increasing awareness that hospital water environment, especially sinks, can be a source of multidrug-resistant bacteria, including CPE, several studies have evaluated methods of sink-trap decontamination, including bleach, alcohol, acetic acid, hot water, hydrogen peroxide vapor, ultraviolet light, and sink-trap replacement.^6,10^ Regev-Yochay et al. attempted to decontaminate sink-traps during an outbreak of OXA-48-producing *Serratia marcescens* by replacing the “disinfecting” sink traps, heating and hyperchlorination of the main water tank, weekly acetic acid treatment, and daily treatment with 1,000 ppm hypochlorite. However, none of aforementioned strategies could disinfect the contaminated sink traps.^11^ In 2019, many sinks in the wards of our hospital were contaminated with CPEs, which prompted us to initiate daily cleaning of the sinks with 1,000 ppm hypochlorite. Although complete elimination was not achieved, the number of CPE-positive sinks was significantly decreased.

Although the replacement of sinks and drainage systems seems to be the most effective method to eliminate CPE,^6^ we could not replace all CPE-positive sinks. In 2021, we replaced sink basins in the PICU, high-care unit, and cardiology wards with deeper ones to facilitate appropriate handwashing procedures and eliminate horizontal transmission of the resistant organisms. Shallow basins can cause cross-contamination of hands during handwashing and promote splashing, with subsequent contamination of the faucet, sink collar, and adjacent surfaces.^12–14^ In 2023, CPEs were not detected from the replaced sinks, and we plan to introduce the deeper basins in the wards other than those mentioned above.

We selected the daily use of 1,000 ppm foam-type sodium hypochlorite to prevent CPE transmission from contaminated sinks because of cost-effectiveness and convenience. According to one review article by Gordon et al. some studies reported successful cessation of CPE outbreaks using disinfection with bleach.^6^ On the other hand, more recently, Buchan et al. reported that a hydrogen peroxide-based foam disinfectant was more effective than liquid bleach in decreasing sink drain bacterial counts.^15^ Furthermore, according to the study of Jones et al. the use of foam-type application of hydrogen peroxide and peracetic acid disinfectant significantly reduced recovery of gram-negative bacilli comparing with pouring liquid.^16^ Although hypochlorite might be less effective than hydrogen peroxide and peracetic acid, daily application of foamy disinfectant was likely effective enough to reduce the number of contaminated sinks.

Although we started cleaning sinks with hypochlorite in 2019, the CPE outbreak in 2020–2021 could not be prevented. Contaminated sinks probably did not play a main role in the outbreak, considering that *E. cloacae* complex was only detected from the sinks in two rooms of the cardiology ward, and eight of the 19 patients became CPE-positive without prior admission to the cardiology ward. At the time of the outbreak, hand hygiene compliance was <70%, and we attributed the outbreak to the low awareness of hand hygiene. To eliminate this outbreak, we enforced infection control methods, including isolation of CPE-positive patients, appropriate use of personal protective equipment, and appropriate handwashing. Since late 2021, hand hygiene at our hospital has remained around 80%.

In our study, the number of CPE-positive sinks was significantly decreased after daily cleaning with bleach was started. However, when data were examined separately for the risk of contact with nutrition-rich substances, only Risk L (lower risk) sinks showed a reduced risk. It is possible that the drain pipes of Risk H (higher risk) sinks contain more biofilms than those of Risk L, leading to the continuous release of resistant bacteria. Parkes et al. reviewed several studies related to strategies for biofilm disruption.^12^ Pressurized steam with a chlorine-containing solution showed temporary clearance followed by re-emergence of carbapenemase-producing *K. pneumoniae*. Self-disinfecting traps, using vibration, heat, and ultraviolet light to prevent biofilm formation, seems promising, although they incur substantial cost and may require further evaluation in healthcare settings.

Considering the difficulties of eliminating CPE-containing biofilm from sink traps, educational interventions seem important. Kotay et al. demonstrated in their experimental sinks where biofilm-containing traps were transplanted that, even if CPE turned negative with soap and water cleaning, they immediately re-emerged after the introduction of carbon- and nitrogen-rich nutrients.^17^ In pediatric wards, excess milk and enteral formula tend to be unintentionally discarded in handwashing sinks. Therefore, strict guidelines regarding sink use should be developed and followed by the staff, including avoiding discarding excess milk into handwashing sinks, specifying sinks that are used exclusively for handwashing, and prohibiting the storage of clean patient material around sinks.

Among the carbapenemases of CPEs, IMP-1 is the most commonly detected in Japan.^17^ Other frequently detected genotypes outside Japan, such as NDM, KPC, and OXA-48, are rarely found in Japan.^18,19^ In our study, the CPEs isolated from sinks and patients had IMP-1 or IMP-11. Although Chen et al. found that IMP-11-producing CRE were reported among the long-term care facilities in Japan,^20^ the Yokohama City Institute of Public Health has never detected CPEs producing IMP-11 except those isolated from our hospital (personal communication). In 2018, three patients were identified as carriers of CPEs producing IMP-11, although none of them were identical species. Therefore, we speculate that these resistant bacteria isolated from patients were likely derived from the sinks, rather than outside the hospital.

This study has several limitations that should be acknowledged. First, we collected environmental samples from only sink bowls and drain outlets. Samples from deeper zone of the drain tubes, faucets, or surfaces outside the sink bowls were not obtained. Furthermore, we seeded samples directly onto selective agar media, without prior broth enrichment. If we obtained samples from wider areas and cultured samples in broth media before seeding onto agar media, CPE might be detected from a higher number of sinks. Second, we obtained environmental culture three times with an interval of 2 years. If we had implemented the surveillance culture more frequently, the effect of our intervention, such as cleaning sink bowls with bleach and prohibiting disposal of milk into sinks, might have been described in more detail. Third, we used PFGE to describe phylogeny of outbreak isolates. Although whole genome sequencing seems to have replaced PFGE in recent outbreak investigations due to its advantage in tracking the outbreak process,^4,21^ our laboratory was in the process of transitioning to a new analysis method such as next-generation sequencing, and we used the conventional analysis method for this study.

In conclusion, after we noticed that three patients admitted to our hospital were CPE-positive, we initiated sequential surveillance cultures of 180 sinks in nine wards of our hospital in 2019, 2021, and 2023. In total, 30 of 180 sinks were contaminated with CPE in 2019. Therefore, we conducted daily cleaning of sinks with sodium hypochlorite. Although the number of CPE-contaminated sinks was significantly decreased, contamination of sinks with a high risk of contact with nutrition-rich substances was not reduced despite daily cleaning with bleach, even after discarding excess milk into the handwashing sinks was prohibited. Because the PFGE patterns of CPEs isolated from patients and nearby sinks were almost identical, it is likely that the contaminated sinks in our hospital wards were reservoirs of disseminating CPE to the patients, although the main cause of the outbreak in 2020–2021 was horizontal transmission due to insufficient hand hygiene. In addition to the efforts to decrease the sink-related CPE contamination, educational interventions, such as compliance monitoring of strict hand hygiene and guidelines of sink use, is important to prevent outbreaks.

## Supporting information

Shikama et al. supplementary material Shikama et al. supplementary material

## Supplementary material

For supplementary material accompanying this paper visit http://doi.org/10.1017/ash.2024.94. click here to view supplementary material
