# Supplementary material for: Carbapenemase-producing Enterobacterales isolated from hospital sinks: molecular relationships with isolates from patients and the change in contamination status after daily disinfection with sodium hypochlorite
Source: Antimicrob Steward Healthc Epidemiol. 2024 Jun 4;4(1):e98. doi: 10.1017/ash.2024.94 (PMC11149038; doi:10.1017/ash.2024.94)
Supplement: Shikama et al. supplementary material [file S2732494X24000949sup001.docx]

Supplementary Table S1. Locations of tested sinks and isolated carbapenemase-producing Enterobacterales (CPE) (species and carbapenemase genotype). Sink bowls in shaded locations were replaced in 2021. PICU: pediatric intensive care unit

| **Sink location** | **Year 2019** | | **Year 2021** | | **Year 2023** | |
| --- | --- | --- | --- | --- | --- | --- |
|  | **Bacteria** | **Carbapenemase** | **Bacteria** | **Carbapenemase** | **Bacteria** | **Carbapenemase** |
| PICU entrance | *Pantoea* sp. | IMP-11 | - | - | - | - |
| PICU milk preparation area | - | - | - | - | - | - |
| PICU parenteral fluid preparation area | - | - | - | - | - | - |
| PICU nurse station 1 | - | - | - | - | - | - |
| PICU nurse station 2 | *Pantoea* sp. | IMP-11 | - | - | - | - |
| PICU nurse station 3 | - | - | - | - | - | - |
| PICU patient room 1 | *Klebsiella oxytoca* | IMP-11 | - | - | - | - |
| PICU patient room 2 | - | - | - | - | - | - |
| PICU patient room 3 | - | - | - | - | - | - |
| High-care patient room 1 | *Pantoea* sp. | IMP-11 | - | - | - | - |
| High-care patient room 2 | *K. oxytoca* | IMP-11 | - | - | - | - |
| High-care patient room 3 | - | - | - | - | - | - |
| High-care patient room 4 | *Enterobacter cloacae* complex | IMP-11 | - | - | - | - |
| High-care milk preparation room | - | - | - | - | - | - |
| High-care parenteral fluid preparation area | *E. cloacae* complex | IMP-11 | *E. cloacae* complex | IMP-11 | - | - |
| High-care nurse station 1 | - | - | - | - | - | - |
| High-care nurse station 2 | - | - | - | - | - | - |
| High-care restroom 1 | - | - | - | - | - | - |
| High-care restroom 2 | - | - | - | - | - | - |
| High-care restroom 3 | - | - | - | - | - | - |
| Cardiology patient room 1 | - | - | - | - | - | - |
| Cardiology patient room 2 | *Pantoea* sp. | IMP-11 | - | - | - | - |
| Cardiology patient room 3 | - | - | - | - | - | - |
| Cardiology patient room 4 | *Pantoea* sp. | IMP-11 | - | - | - | - |
| Cardiology patient room 5 | *Pantoea* sp. | IMP-11 | *E. cloacae* complex | IMP-1 | - | - |
| Cardiology patient room 6 | *Pantoea* sp. | IMP-11 | *E. cloacae* complex | IMP-1 | - | - |
| Cardiology patient room 7 | *Pantoea* sp. | IMP-11 | - | - | - | - |
| Cardiology patient room 8 | - | - | - | - | - | - |
| Cardiology patient room 9-1 | - | - | - | - | - | - |
| Cardiology patient room 9-2 | - | - | - | - | - | - |
| Cardiology playroom | *Pantoea* sp. | IMP-11 | - | - | - | - |
| Cardiology treatment room | *Pantoea* sp. | IMP-11 | - | - | - | - |
| Cardiology dish washing sink | - | - | - | - | - | - |
| Cardiology milk preparation room 1 | - | - | - | - | - | - |
| Cardiology milk preparation room 2 | - | - | - | - | - | - |
| 4W patient room 1 | - | - | - | - | - | - |
| 4W patient room 2 | - | - | - | - | - | - |
| 4W patient room 3 | - | - | - | - | - | - |
| 4W patient room 4 | - | - | - | - | - | - |
| 4W patient room 5 | - | - | - | - | - | - |
| 4W patient room 6 | - | - | - | - | - | - |
| 4W patient room 7 | - | - | - | - | - | - |
| 4W patient room 8 | - | - | - | - | - | - |
| 4W patient room 9 | - | - | - | - | - | - |
| 4W patient room 10 | - | - | - | - | - | - |
| 4W patient room 11 | *Pantoea* sp. | IMP-11 | - | - | - | - |
| 4W patient room 12 | - | - | - | - | - | - |
| 4W restroom 1 | - | - | - | - | - | - |
| 4W restroom 2 | - | - | - | - | - | - |
| 4W restroom 3 | - | - | - | - | - | - |
| 4W milk preparation area 1 | - | - | - | - | - | - |
| 4W milk preparation area 2 | - | - | - | - | - | - |
| 4W treatment room 1 | - | - | - | - | - | - |
| 4W treatment room 2 | - | - | - | - | - | - |
| 4W treatment room 3 | - | - | - | - | - | - |
| 4W nurse station 1 | - | - | - | - | - | - |
| 4W nurse station 2 | - | - | - | - | - | - |
| 4W dirty utility room | - | - | - | - | - | - |
| 4W parenteral fluid preparation area | *E. cloacae* complex | IMP-11 | *E. cloacae* complex | IMP-11 | - | - |
| 4W playroom | - | - | - | - | - | - |
| 4S patient room 1 | - | - | - | - | - | - |
| 4S patient room 2 | - | - | - | - | - | - |
| 4S patient room 3 | - | - | - | - | - | - |
| 4S patient room 4 | - | - | - | - | - | - |
| 4S patient room 5 | - | - | - | - | - | - |
| 4S patient room 6 | - | - | - | - | - | - |
| 4S patient room 7 | - | - | - | - | - | - |
| 4S patient room 8 | - | - | - | - | - | - |
| 4S patient room 9 | - | - | - | - | - | - |
| 4S patient room 10 | - | - | - | - | - | - |
| 4S patient room 11 | - | - | - | - | - | - |
| 4S patient room 12 | - | - | - | - | - | - |
| 4S nurse station 1 | *Pantoea* sp. | IMP-11 | - | - | - | - |
| 4S nurse station 2 | - | - | - | - | - | - |
| 4S treatment room 1 | - | - | - | - | - | - |
| 4S treatment room 2 | - | - | - | - | - | - |
| 4S treatment room 3 | - | - | - | - | - | - |
| 4S milk preparation area 1 | - | - | - | - | - | - |
| 4S milk preparation area 2 | - | - | - | - | - | - |
| 4S restroom 1 | - | - | - | - | - | - |
| 4S restroom 2 | - | - | - | - | - | - |
| 4S restroom 3 | - | - | - | - | - | - |
| 4S parenteral fluid preparation area | *Citrobacter freundii* complex | IMP-11 | *C. freundii* complex | IMP-11 | - | - |
| 4S dirty utility room | - | - | - | - | - | - |
| 4S playroom | - | - | - | - | - | - |
| 4E parenteral fluid preparation area | - | - | - | - | *K. oxytoca* | IMP-11 |
| 4E milk preparation area 1 | *Pseudescherichia vulneris* | IMP-1 | *K. oxytoca* | IMP-11 | - | - |
| 4E milk preparation area 2 | *P. vulneris* | IMP-1 | - | - | *E. cloacae* complex | IMP-11 |
| 4E nurse station 1 | *Pantoea* sp. | IMP-11 | *K. oxytoca* | IMP-11 | - | - |
| 4E nurse station 2 | - | - | - | - | - | - |
| 4E treatment room | - | - | - | - | - | - |
| 4E patient room 1 | - | - | - | - | - | - |
| 4E patient room 2 | - | - | - | - | - | - |
| 4E patient room 3 | - | - | - | - | - | - |
| 4E patient room 4 | - | - | - | - | - | - |
| 4E patient room 5 | - | - | - | - | - | - |
| 4E patient room 6 | *Pantoea* sp. | IMP-11 | - | - | - | - |
| 4E patient room 7 | - | - | - | - | - | - |
| 4E patient room 8 | - | - | *K. oxytoca* | IMP-11 | - | - |
| 4E patient room 9 | - | - | - | - | - | - |
| 4E patient room 10 | - | - | *Pantoea* sp. | IMP-11 | - | - |
| 4E patient room 11 | - | - | - | - | - | - |
| 4E patient room 12 | - | - | - | - | - | - |
| 5W patient room 1-1 | - | - | - | - | - | - |
| 5W patient room 1-2 | - | - | - | - | - | - |
| 5W patient room 2-1 | - | - | - | - | - | - |
| 5W patient room 2-2 | - | - | - | - | - | - |
| 5W patient room 3 | - | - | - | - | - | - |
| 5W patient room 4 | - | - | - | - | - | - |
| 5W patient room 5 | - | - | - | - | - | - |
| 5W patient room 6 | - | - | - | - | - | - |
| 5W patient room 7 | - | - | - | - | - | - |
| 5W patient room 8 | - | - | - | - | - | - |
| 5W patient room 9 | *E. cloacae* complex | IMP-11 | - | - | - | - |
| 5W patient room 10 | - | - | - | - | - | - |
| 5W patient room 11 | - | - | *C. freundii* complex | IMP-11 | - | - |
| 5W patient room 12 | - | - | - | - | - | - |
| 5W nurse station 1 | - | - | - | - | - | - |
| 5W nurse station 2 | - | - | - | - | - | - |
| 5W milk preparation area 1 | - | - | - | - | - | - |
| 5W milk preparation area 2 | - | - | - | - | - | - |
| 5W parenteral fluid preparation area | *Pantoea* sp. | IMP-11 | *C. freundii* complex | IMP-11 | - | - |
| 5W restroom 1 | - | - | - | - | *E. cloacae* complex | IMP-1 |
| 5W restroom 2 | - | - | - | - | *E. cloacae* complex | IMP-1 |
| 5W restroom 3 | - | - | - | - | *E. cloacae* complex | IMP-1 |
| 5W playroom | - | - | - | - | - | - |
| 5W dish washing sink | - | - | - | - | - | - |
| 5W treating room 1 | - | - | - | - | - | - |
| 5W treating room 2 | - | - | - | - | - | - |
| 5W dirty utility room | - | - | - | - | - | - |
| 5S patient room 1-1 | - | - | - | - | - | - |
| 5S patient room 1-2 | - | - | - | - | - | - |
| 5S patient room 2-1 | - | - | - | - | - | - |
| 5S patient room 2-2 | - | - | - | - | - | - |
| 5S patient room 3 | - | - | - | - | - | - |
| 5S patient room 4 | - | - | - | - | - | - |
| 5S patient room 5 | - | - | - | - | - | - |
| 5S patient room 6 | *Pantoea* sp. | IMP-11 | - | - | - | - |
| 5S patient room 7 | - | - | - | - | - | - |
| 5S patient room 8 | - | - | - | - | - | - |
| 5S patient room 9 | *Pantoea* sp. | IMP-11 | - | - | - | - |
| 5S patient room 10 | *Pantoea* sp. | IMP-11 | - | - | - | - |
| 5S patient room 11 | *Pantoea* sp. | IMP-11 | - | - | - | - |
| 5S patient room 12 | - | - | - | - | - | - |
| 5S playroom 1 | - | - | - | - | - | - |
| 5S playroom 2 | - | - | - | - | - | - |
| 5S nurse station | *Pantoea* sp. | IMP-11 | - | - | - | - |
| 5S parenteral fluid preparation area | *E. cloacae* complex | IMP-1 | *E. cloacae* complex | IMP-1 | - | - |
| 5S treatment room 1 | - | - | - | - | - | - |
| 5S treatment room 2 | - | - | - | - | - | - |
| 5E patient room 1-1 | - | - | - | - | - | - |
| 5E patient room 1-2 | - | - | - | - | - | - |
| 5E patient room 2-1 | - | - | - | - | - | - |
| 5E patient room 2-2 | - | - | - | - | - | - |
| 5E patient room 3-1 | - | - | - | - | - | - |
| 5E patient room 3-2 | - | - | - | - | - | - |
| 5E patient room 4-1 | - | - | - | - | - | - |
| 5E patient room 4-2 | - | - | - | - | - | - |
| 5E patient room 5 | - | - | - | - | - | - |
| 5E patient room 6-1 | - | - | - | - | - | - |
| 5E patient room 6-2 | - | - | - | - | - | - |
| 5E patient room 7 | - | - | - | - | - | - |
| 5E patient room 8 | - | - | - | - | - | - |
| 5E patient room 9 | - | - | - | - | - | - |
| 5E patient room 10 | - | - | - | - | - | - |
| 5E patient room 11 | - | - | - | - | - | - |
| 5E restroom 1 | - | - | - | - | - | - |
| 5E restroom 2 | - | - | - | - | - | - |
| 5E restroom 3 | - | - | - | - | - | - |
| 5E treatment room | - | - | - | - | - | - |
| 5E dirty utility room | - | - | - | - | - | - |
| 5E playroom | - | - | - | - | - | - |
| 5E milk preparation area | - | - | - | - | - | - |
| 5E bathroom | - | - | - | - | - | - |
| 5E nurse station 1 | - | - | - | - | - | - |
| 5E nurse station 2 | - | - | - | - | - | - |
| 5E parenteral fluid preparation area | - | - | *E. cloacae* complex | IMP-1 | *E. cloacae* complex | IMP-1 |
| 5E entrance | - | - | - | - | - | - |
